# Clinical advances in the pharmacotherapy of congenital adrenal hyperplasia

**DOI:** 10.1530/EJE-21-0794

**Published:** 2021-11-04

**Authors:** Alessandro Prete, Richard J Auchus, Richard J Ross

**Affiliations:** 1 Institute of Metabolism and Systems Research, University of Birmingham, Birmingham, UK; 2 Department of Endocrinology, Queen Elizabeth Hospital Birmingham, University Hospitals Birmingham NHS Foundation Trust, Birmingham, UK; 3 Division of Metabolism, Endocrinology and Diabetes, Departments of Pharmacology and Internal Medicine, University of Michigan, Ann Arbor, Michigan, USA; 4 Department of Oncology and Metabolism, University of Sheffield, Sheffield, UK

## Abstract

**Background:**

Patients with 21-hydroxylase deficiency congenital adrenal hyperplasia (21OHD-CAH) have poor health outcomes with increased mortality, short stature, impaired fertility, and increased cardiovascular risk factors such as obesity. To address this, there are therapies in development that target the clinical goal of treatment, which is to control excess androgens with an adrenal replacement dose of glucocorticoid.

**Methods:**

Narrative review of publications on recent clinical developments in the pharmacotherapy of congenital adrenal hyperplasia.

**Summary:**

Therapies in clinical development target different levels of the hypothalamo–pituitary–adrenal axis. Two corticotrophin-releasing factor type 1 (CRF_1_) receptor antagonists, Crinecerfont and Tildacerfont, have been trialled in poorly controlled 21OHD-CAH patients, and both reduced ACTH and androgen biomarkers while patients were on stable glucocorticoid replacement. Improvements in glucocorticoid replacement include replacing the circadian rhythm of cortisol that has been trialled with continuous s.c. infusion of hydrocortisone and Chronocort, a delayed-release hydrocortisone formulation. Chronocort optimally controlled 21OHD-CAH in 80% of patients on an adrenal replacement dose of hydrocortisone, which was associated with patient-reported benefits including restoration of menses and pregnancies. Adrenal-targeted therapies include the steroidogenesis-blocking drug Abiraterone acetate, which reduced adrenal androgen biomarkers in poorly controlled patients.

**Conclusions:**

CRF_1_ receptor antagonists hold promise to avoid excess glucocorticoid replacement in patients not controlled on standard or circadian glucocorticoid replacement such as Chronocort. Gene and cell therapies are the only therapeutic approaches that could potentially correct both cortisol deficiency and androgen excess.

## Background

The discovery of cortisone – and its use as replacement therapy for adrenal insufficiency – was life-saving for patients born with classic congenital adrenal hyperplasia (CAH) (1). Since the 1960s, however, there has been little innovation in the pharmacological treatment of patients with classic CAH, due to 21-hydroxylase deficiency (21OHD-CAH), and in the last decade, cohort studies have reported poor health outcomes in adults with 21OHD-CAH (2, 3, 4, 5). These observational studies have stimulated the development of new pharmacotherapies to reduce the morbidity and mortality of patients with 21OHD-CAH. This review concentrates on the therapies that are now in clinical development.

**Figure 1:**
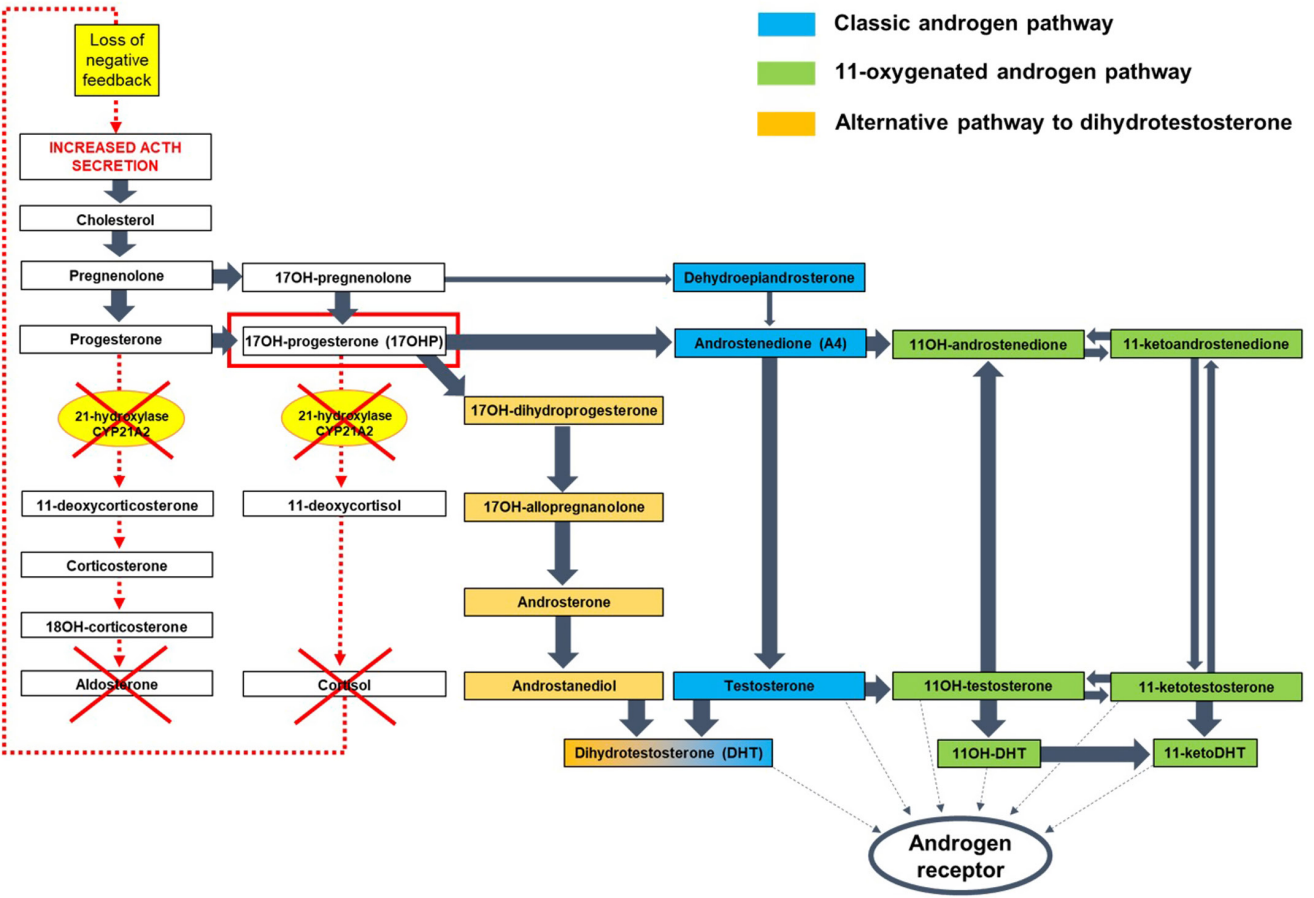
Pathophysiology of CAH due to 21-hydroxylase deficiency. Simplified representation of the steroid hormone biosynthesis, with a focus on androgen generation. 21-hydroxylase deficiency causes defective secretion of aldosterone and cortisol. The latter leads to excessive ACTH secretion from the pituitary, which results in adrenal androgen excess. Androgens can be generated through three pathways: the classic androgen pathway through DHEA; the 11-oxygenated androgen pathway through androstenedione (A4); and the alternative pathway to dihydrotestosterone (DHT) through androsterone. The alternative DHT pathway is active in the testis during male development in the foetus but not active in childhood and adults. The accumulation of 17OH-progesterone (17OHP) in CAH (circled in red) increases atypical conversion of 17OHP to A4 by CYP17A1 17,20-lyase activity, which physiologically has a much higher preference for the conversion of 17OH-pregnenolone to DHEA. Accumulating 17OHP also drives increased androgen production by the alternative DHT pathway, and increased A4 feeds enhanced 11-oxygenated androgen pathway activity. The classic pathway via DHEA is downregulated in CAH.

**Figure 2:**
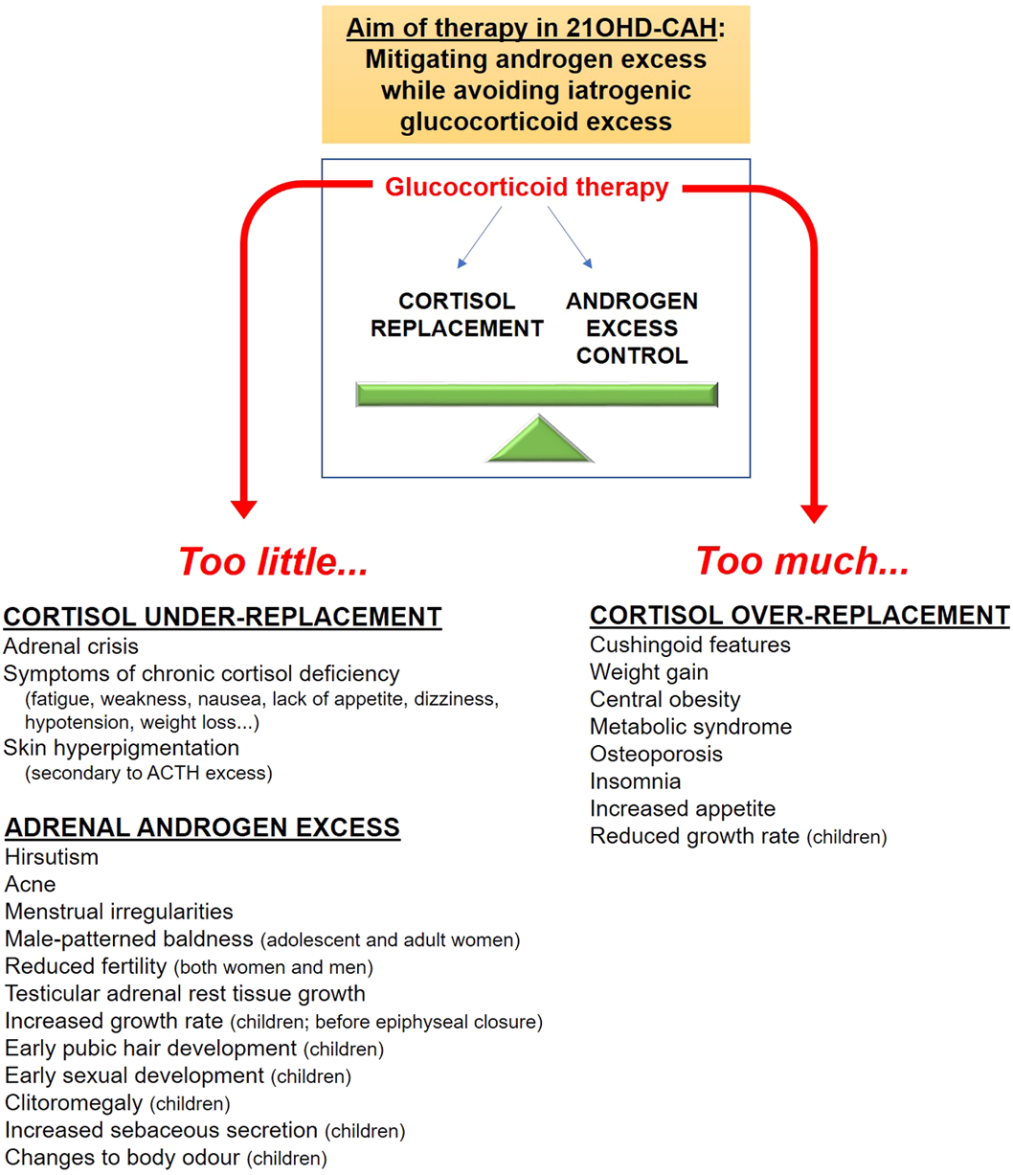
Challenges of CAH treatment. The current standard of care for patients with CAH is glucocorticoid therapy, which targets both the cortisol deficiency and the adrenal androgen excess.

21OHD-CAH is a genetic disorder of steroidogenesis affecting ~1:15 000 live births (5). Lack of 21-hydroxylase causes cortisol deficiency and a counter-regulatory increase in pituitary adrenocorticotropic hormone (ACTH) secretion, which drives the overproduction of adrenal androgens, and adrenal hyperplasia (Fig. 1). Patients with 21OHD-CAH have two major problems: adrenal insufficiency and androgen excess. Adrenal insufficiency causes life-threatening adrenal crises (5, 6, 7), while androgen excess causes atypical genitalia in 46,XX neonates, promotes abnormal growth, culminates in short adult stature, sometimes triggers precocious puberty, and in adulthood, virilization of women and infertility in both sexes (8). Treatment aims are to replace cortisol and, where required, aldosterone. Supraphysiologic doses of glucocorticoid are typically needed to lower ACTH and adrenal androgens, which chronically exposes patients to excess glucocorticoid treatment over time. Management of 21OHD-CAH involves balancing glucocorticoid doses to avoid glucocorticoid deficiency, risking adrenal crisis, and iatrogenic glucocorticoid excess, which predisposes to short stature, obesity, hypertension, osteoporosis, and an adverse metabolic profile (Fig. 2) (4, 5, 6, 7, 9, 10).

Patients with 21OHD-CAH have increased mortality up to five times that of the healthy population (11, 12), with adrenal crisis the leading cause of death (12). Patients with 21OHD-CAH have increased cardiovascular disease risk factors and metabolic morbidity as shown in the National Institute of Health natural history study published in 2021 (13). This is the largest longitudinal study in 21OHD-CAH with patients followed from childhood with a median follow-up 18.6 years; doses of hydrocortisone at the beginning and end of the study were 17.7 and 17.4 mg/m^2^/day, respectively, and exceeded those recommended for adrenal replacement of 8–14 mg/m^2^/day (14, 15); adrenal androgens were only considered controlled at 28% of visits; 93% of patients were hypertensive at one or more visits; obesity was prevalent and increasing from childhood with 56% children 49% adults obese; and insulin resistance was observed in 94.7% patients at one or more visits. Adults with 21OHD-CAH are approximately 10 cm shorter than the population mean and have a higher prevalence of anxiety, depression, alcohol misuse, and personality disorders, with suicides among male patients and adjustment disorders among female patients (5). Fertility is reduced in female and male adults with 21OHD-CAH. In women, androgen hypersecretion results in anovulation, and acyclic progesterone hypersecretion acts like a progestin-only contraceptive. Oligomenorrhoea and/or amenorrhoea are reported in 45% of women with 21OHD-CAH compared to 13.6% in the healthy population (2, 16), and women with 21OHD-CAH report only 0.25 live births per woman compared with 1.8 in the UK population; however, fecundity is normal in those wishing to get pregnant and receiving proper treatment (17). In men, ACTH excess drives the development of testicular adrenal rest tissue, which over time results in fibrosis and azoospermia, and adrenal-derived androgen hypersecretion suppresses the pituitary–gonadal axis. In the UK cohort study, 37% of males had sought fertility support, of which only 67% were successful (2). A separate study of 50 male clinic attendees with 21OHD-CAH reported severe oligospermia in 48% (18). In both women and men with 21OHD-CAH, fertility can be improved with supraphysiological doses of glucocorticoid, but this approach has the downside of all the complications from excess glucocorticoid exposure.

The majority of the poor health outcomes in patients with 21OHD-CAH result from the inability to precisely titrate currently available glucocorticoid preparations to both adequately replace the deficiency and sufficiently attenuate the adrenal-derived androgen excess. Undertreatment causes an increased risk of adrenal crisis and poor androgen control, and overtreatment causes iatrogenic Cushing’s syndrome. To understand the pathology of 21OHD-CAH and the impact of new pharmacotherapies on disease biomarkers, we need to understand how adrenal androgens are generated in patients with 21OHD-CAH (Fig. 1). In normal physiology, there are essentially three pathways to adrenal androgen production: the classic via DHEA, the 11-oxygenated via androstenedione (A4), and the alternative dihydrotestosterone (DHT) pathway via 17-hydroxyprogesterone (17OHP) that is usually only active in the foetus (19). In patients with 21OHD-CAH, these pathways are deranged; the classic pathway via DHEA is downregulated, and DHEA levels are normal or low, although the mechanism for this is not understood. With the exaggerated ACTH drive, 17OHP levels are elevated, and 17OHP is a key intermediate driving the three androgen pathways to testosterone and 11-oxygenated androgens via A4 and by activating the normally quiescent alternative DHT pathway. 17OHP is therefore a key biomarker for assessing disease control in 21OHD-CAH. 17OHP levels in healthy individuals have a minimal circadian rhythm, and although there is some variation by gender and during the menstrual cycle, the upper limit of normal (ULN) for 17OHP levels is ~12 nmol/L (400 ng/dL) in most quoted references ranges. In 21OHD-CAH, 17OHP levels can be very high in the early morning, often >50 nmol/L (1650 ng/dL), and it is recognized that to control 17OHP in to the reference range on standard glucocorticoid therapy risks overtreatment (20). For this reason, endocrinologists have adopted a target optimal range for 17OHP < 3× ULN ~ 36 nmol/L (1200 ng/dL); patients having a morning 17OHP < 36 nmol/L (1200 ng/dL) are considered to have good disease control (2, 3). This criterion is an important concept when we come to assess new therapies.

**Figure 3:**
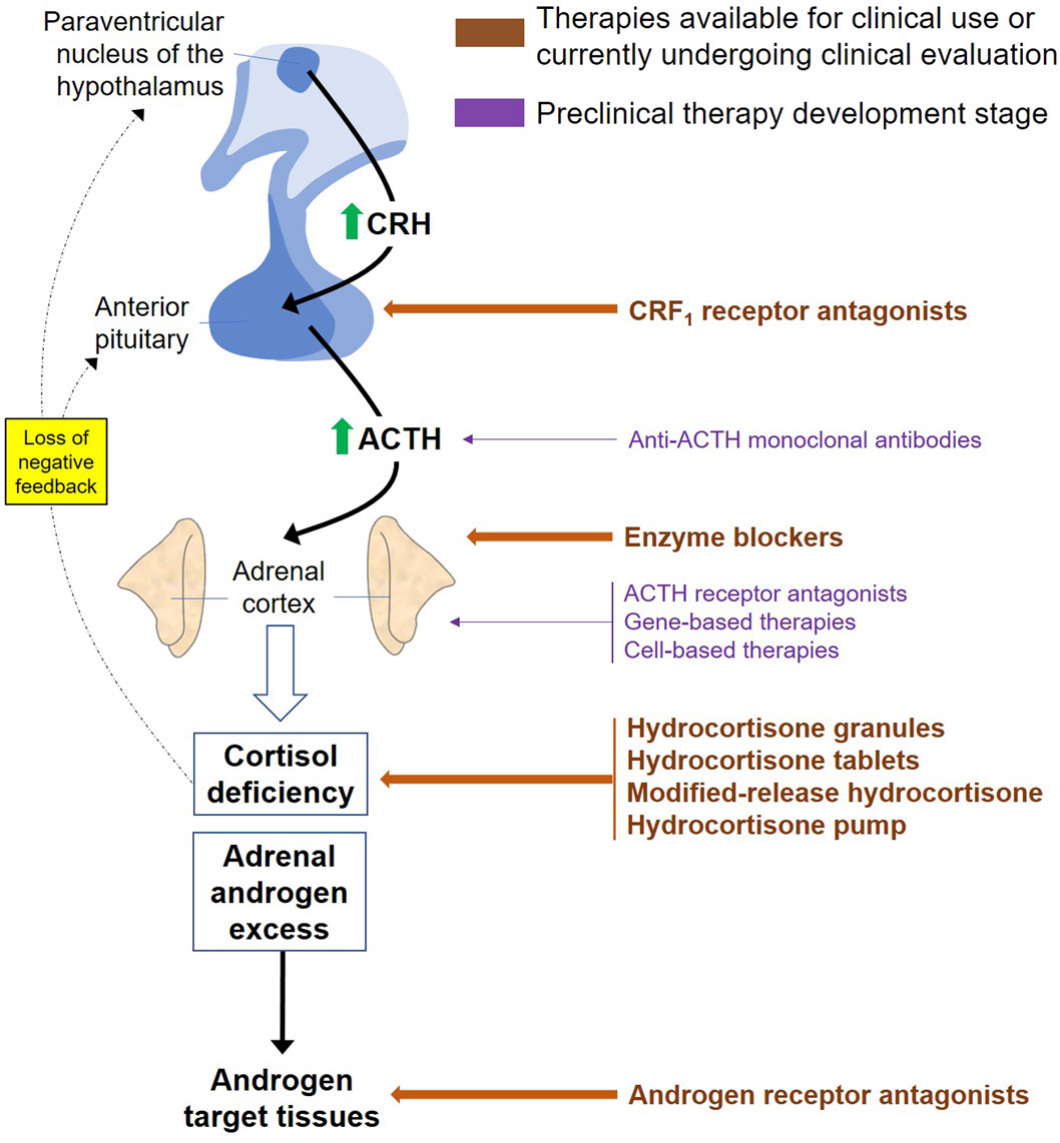
Pharmacotherapies for CAH target different levels of the HPA axis. The hypothalamus controls ACTH release from the pituitary through CRF, and ACTH in turn stimulates cortisol release from the adrenal that feeds back at the hypothalamus and pituitary in a classic endocrine feedback loop.

To address the poor health outcomes and unmet need for better treatment of 21OHD-CAH, researchers have targeted different levels of the hypothalamic–pituitary–adrenal axis from suppressing ACTH release from the pituitary to gene therapy at the adrenal (Fig. 3). In this review, we report on the current status of the evolving pharmacotherapies for 21OHD-CAH that are being trialled in the clinic (Table 1).

**Table 1:** Novel treatment strategies for congenital adrenal hyperplasia that are available for clinical use or currently undergoing clinical evaluation.

Treatment	Administration	Advantages	Disadvantages
CRF1 receptor antagonists			
Crinecerfont*	Oral, twice daily.	•Oral administration. • Effectively reduced ACTH and adrenal androgen • Possibly leads to reduced glucocorticoid requirements. • Favourable safety profile	• Lack of long-term efficacy and safety data. • Need for concomitant glucocorticoid replacement.
Tildacerfont*	Oral, once daily.	• Oral administration. • Effectively reduces ACTH and adrenal androgen levels. • Possibly leads to reduced glucocorticoid requirements. • Favourable safety profile. • Associated with testicular adrenal rest tissue reduction.	• Lack of long-term efficacy and safety data. • Need for concomitant glucocorticoid replacement. • Drug–drug interactions (including increased bioavailability of dexamethasone).
Glucocorticoid replacement			
Modified-release hydrocortisone Plenadren^®^	Oral, once daily.	• Oral administration. • Once daily dosing. • Favourable safety profile.	• Reduced bioavailability • Does not replace overnight cortisol levels.
Modified-release hydrocortisone (Efmody^®^; development name Chronocort)	• Oral, twice daily. • Recommended starting dose: 10–15 mg/m^2^/day (adolescents ≥ 12 years who have not completed growth); 15–25 mg/day (adults and adolescents who have completed growth). • The starting dose should be split into ⅔–¾ given in the evening at bedtime and the rest given in the morning upon awakening.	• Oral administration. • Mimics the natural cortisol rhythm. • Effectively controls androgen excess. • Associated with clinical benefit (restoration of menses, partner pregnancies, improvement of sperm count in a man with testicular adrenal rest tissue). • Favourable safety profile.	• Cannot be used for sick day dosing (patients should be provided with immediate-release glucocorticoid formulations, for example, hydrocortisone or cortisone acetate). • Not recommended in patients with increased gastrointestinal motility due to the risk of impaired absorption.
Hydrocortisone pump	Continuous s.c. infusion.	• Mimics the natural cortisol rhythm. • Effectively controls androgen excess. • Associated with clinical benefit (quality of life, restoration of menses, testicular adrenal rest tissue reduction) • Relies on technology that is already available (insulin pump).	• Evidence derived from one small-scale study. • Requires continuous device wear and high engagement by the patient and health professionals. • Possibility of malfunction. • Available hydrocortisone formulations are not designed for s.c. use. • Possible injection site and systemic reactions.
Alkindi^®^ hydrocortisone granules (development name Infacort)	Oral, two to four times daily	• Oral administration. • Paediatric appropriate dosing available as 0.5, 1.0, 2.0, and 5 mg. • Taste masking. • Favourable safety profile.	• Does not replace overnight cortisol levels.
Acecort^®^ hydrocortisone tablets	Oral, two to four times daily	• Oral administration. • Dosing available at 1.0, 5.0, and 10 mg. • Colour-coded	• Does not replace overnight cortisol levels.
CYP17A1 inhibitor			
Abiraterone acetate*	Oral, once daily.	• Oral administration. • Effectively controls androgen excess. • Favourable safety profile.	• Need for concomitant glucocorticoid replacement. • Inhibits gonadal sex steroid biosynthesis and could be used only in patients who do not desire fertility. • Potential adverse foetal outcomes. • Potential concern for expansion of testicular adrenal rest tissue in men. • Concerns around liver toxicity. • Needs to be taken on an empty stomach (at least 1 h before and 2 h after any food). • Drug–drug interactions.
Androgen receptor antagonist			
Flutamide*	Oral, three times daily.	• Oral administration. • Associated with normal linear growth and reduced glucocorticoid requirements in a small-scale study.	• Need for concomitant glucocorticoid ± aromatase inhibitor treatment. • Concerns around liver toxicity. • Contraception is recommended in women of reproductive age.

*Currently used in patients with 21OHD-CAH only as part of clinical trials.

## Corticotrophin-releasing factor receptor antagonists

Elevated ACTH is the primary driver for adrenal androgen production in 21OHD-CAH, and therefore, suppressing ACTH release is a rational approach to therapy in 21OHD-CAH. The primary regulator of ACTH synthesis and release is corticotrophin-releasing factor (CRF), released from the hypothalamus into the hypophyseal portal system, acting directly on specific receptors on pituitary corticotropes. Two different types of CRF receptors exist: the CRF type 1 receptor (CRF_1_), abundant in the pituitary, and the CRF type 2 receptor (CRF_2_), predominantly found in peripheral tissues (21). Small-molecule CRF1 receptor antagonists have been synthesized and tested in patients with 21OHD-CAH (22), and two orally active, selective, non-steroidal CRF1 receptor antagonists are in development as therapies for 21OHD-CAH: Crinecerfont (Neurocrine Biosciences, Inc, USA) and Tildacerfont (Spruce Biosciences, USA).

**Figure 4:**
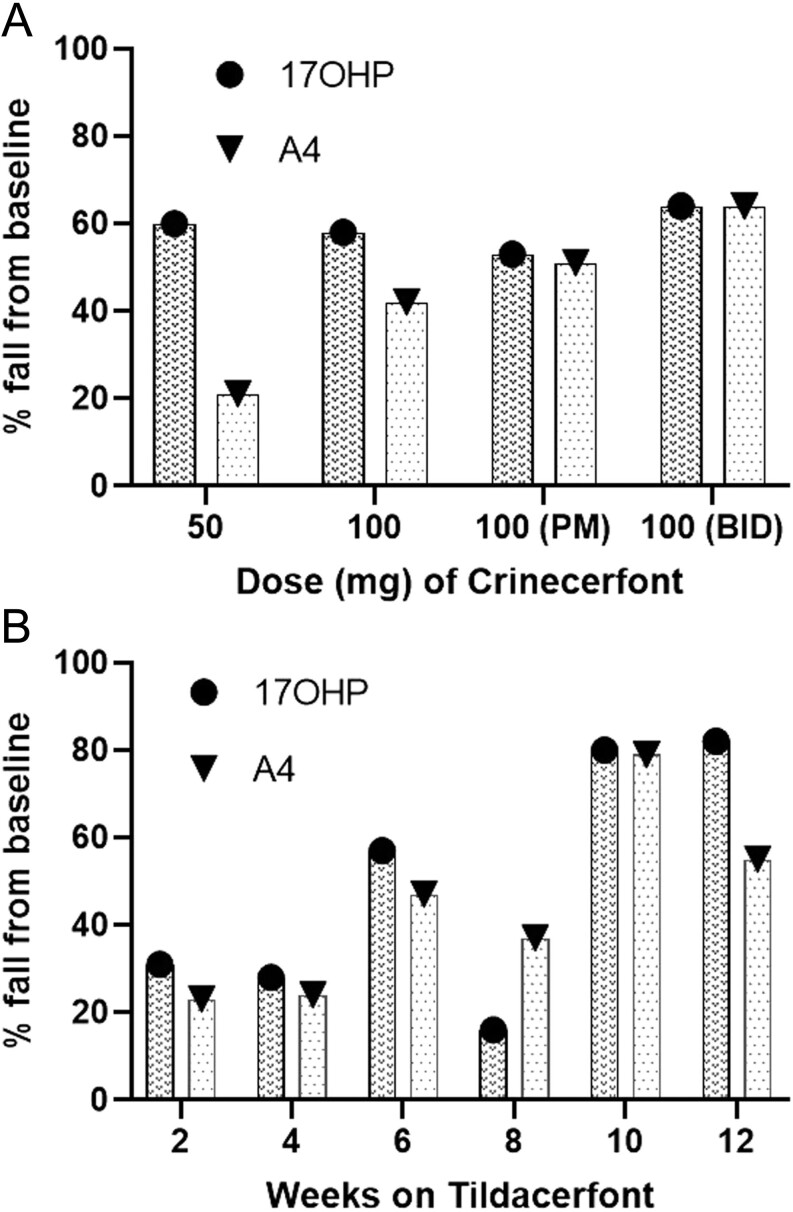
Effect of treatment with CRF1 receptor antagonists on CAH biomarkers. (A) Percentage fall in 17OHP and androstenedione (A4) after 14 days of Crinecerfont oral dosing: 50 mg at bedtime (*n* = 8), 100 mg at bedtime (*n* = 7), 100 mg once daily with an evening meal (*n* = 8), and 100 mg twice daily with meals (*n* = 8). (B) Percentage fall in 17OHP and A4 during Tildacerfont treatment in patients with poor control of CAH at baseline.

The development of Crinecerfont was based on a phase Ib study of NBI-77860, another CRF1 receptor antagonist, which afforded dose-dependent reductions of ACTH and 17OHP in a single-dose study of 300 and 600 mg at bedtime (22). The phase 2 open-label, multiple dose, dose-finding study evaluated four Crinecerfont oral dosing regimens administered for 14 consecutive days: 50 mg at bedtime (*n* = 8), 100 mg at bedtime (*n* = 7), 100 mg once daily with an evening meal (*n* = 8), and 100 mg twice daily with meals (*n* = 8) (23). The median daily baseline glucocorticoid dose was ~25 mg hydrocortisone dose equivalents, and patients were generally poorly controlled with the median baseline morning 17OHP ranging from 5000 to 12 800 ng/dL (152–389 nmol/L) across the groups. Crinecerfont treatment delayed and attenuated the morning ACTH rise and produced meaningful reductions in ACTH and 17-OHP levels by 54–75% at all doses studied, but the median 17OHP remained above 2000 ng/dL (60 nmol/L) in all groups (Fig. 4A). There was a dose-related decrease in A4 levels, ranging from 21 to 64%, and in three of the four cohorts, the A4 median fell in the normal range. The reduced adrenal androgen production induced by Crinecerfont was also documented by a fall of testosterone levels in women and of the A4/testosterone ratio in men ranging from 32 to 74% and from 33 to 59%, respectively. Treatment with Crinecerfont was well tolerated with a favourable safety profile with no related severe adverse events reported. Two ongoing phase 3 trials are assessing the long-term efficacy and safety of Crinecerfont in both adult and paediatric patients with 21OHD-CAH (NCT04490915 and NCT04806451).

Two phase 2 studies have assessed the efficacy and safety of Tildacerfont. Adults with 21OHD-CAH receiving stable glucocorticoid replacement and 17OHP at baseline ≥800 ng/dL (24 nmol/L) were treated with oral Tildacerfont 200–1000 mg once daily (*n* = 10) or 100–200 mg twice daily (*n* = 9) for 2 weeks (study 1) and 400 mg once daily (*n* = 11) for 12 weeks (study 2) (24). In study 1, participants with poor disease control at baseline (defined as A4 > 2× ULN) had reductions in ACTH (−59.4 to −28.4%), 17-OHP (−38.3 to 0.3%), and A4 (−24.2 to −18.1%), with no dose-response relationship. In study 2, patients with poor disease control at baseline achieved ~80% maximum mean reductions in ACTH, 17OHP, and A4 (Fig. 4B). ACTH normalized in 60% of participants and A4 in 40%. In both studies, participants with good disease control at baseline (A4 ≤ 2× ULN) showed only minor changes in ACTH, 17OHP, and A4 levels. Volume reduction of testicular adrenal rest tissue was observed in two out of three male participants who had post-treatment testicular ultrasounds. Adverse events were generally mild, and one participant discontinued Tildacerfont due to grade 1 pruritus with grade 1 elevations in ALT/AST (<3× ULN) with no change to direct bilirubin. Of note, a CYP3A4-mediated interaction between Tildacerfont and dexamethasone was identified, resulting in approximately a two-fold increased dexamethasone exposure. This finding is relevant as dexamethasone is commonly used in patients with 21OHD-CAH, and a switch to a different glucocorticoid may be required before starting Tildacerfont treatment. Currently, two phase 2b studies (NCT04457336, NCT04544410) are testing whether treatment with Tildacerfont can achieve long-lasting clinical benefit and allow reduction of the glucocorticoid dose while controlling relevant disease biomarkers.

In summary, the available evidence on CRF1 receptor antagonists shows that they can attenuate the exaggerated morning rise in ACTH in poorly controlled patients with 21OHD-CAH. The reduction of morning ACTH is associated with a fall in the key biomarkers of disease control, 17OHP and A4. Future studies of CRF1 receptor antagonists are now being planned to examine their clinical benefit in patients with 21OHD-CAH.

## Optimized hydrocortisone dosing in children

The commonest cause of adrenal insufficiency (AI) in young children is CAH (25). The recommended therapy in childhood is hydrocortisone given three to four times daily (26). Until 2018, the lowest available licenced preparations of hydrocortisone were 10 mg tablets in Europe and 5 mg tablets in the United States (US). As scored tablets are licenced to be divided into halves, the lowest possible available dose was 5 mg (Europe) and 2.5 mg (US), respectively. However, these doses are not appropriate to treat neonates, infants, and young children with adrenal insufficiency who require a daily dose of 10–15 mg/m² with single doses as low as 0.5 mg (27). Crushed hydrocortisone tablets suspended in water are often used in some countries (26), though accurate dosing is not possible as hydrocortisone does not dissolve well in water and may adhere to plastic material when applied with syringes (28, 29). Another common practice in pharmacies is to compound hydrocortisone often mixed with sucrose to overcome the inherent bitterness of hydrocortisone. However, a German study demonstrated that up to 25% of compounded batches do not fulfil the acceptance criteria of the European Pharmacopeia in uniformity of net mass or drug content or are labelled inaccurately (30). In Europe, UK, and US, Alkindi^®^ hydrocortisone granules (development name Infacort, Diurnal Europe B.V., The Netherlands) have now become licenced for children with AI from birth to 18 years of age and are available in low doses of 0.5, 1, 2, and 5 mg. They were developed to address the age group-specific needs of neonates, infants, and young children (31, 32). As part of the development programme, a single-dose clinical trial was undertaken in neonates, infants, and children under 6 years with AI, the majority of whom had CAH (33). The children were then invited to participate in a prospective follow-up study of continued treatment with hydrocortisone granules (34). Seventeen children with CAH aged from birth to 6 years had their hydrocortisone medication changed from pharmacy compounded capsules to hydrocortisone granules. Patients were followed prospectively for 2 years. Median daily hydrocortisone dose varied with age groups and declined from entry to end of study: 9.9–12.0 to 8.6–10.2 mg/m^2^/day, respectively. There were no trends for accelerated or reduced growth. No adrenal crises were observed despite 193 treatment-emergent adverse events, which were mainly common childhood illnesses. This first prospective study of glucocorticoid treatment in children with AI and CAH demonstrated that accurate dosing and monitoring from birth results in hydrocortisone doses at the lower end of the recommended dose range, normal growth, without the occurrence of adrenal crises. Hydrocortisone tablets at 1, 5, and 10 mg are available in the Netherlands (Acecort®, hydrocortisone tablets, ACE Pharmaceuticals B.V.). The Netherlands public assessment report states that Acecort is indicated for the treatment of AI in patients in which other hydrocortisone-containing products (prolonged release) cannot be used and/or in case of excessive physical and/or mental stress (adrenal crisis). There are no published studies of Acecort® in children with CAH.

## Circadian glucocorticoid therapies

ACTH rises overnight from around 2:00–4:00 h to provide the circadian rhythm of cortisol that peaks shortly after waking and falls to low levels in the evening (35). This rise in ACTH is exaggerated in 21OHD-CAH because of the failure in cortisol negative feedback as seen in children taking hydrocortisone (36). The issue being that hydrocortisone has a short plasma half-life, such that even when a dose is taken in the evening, cortisol levels are low before the morning rise in ACTH (37). To address this consideration, clinicians have used a number of approaches including reverse circadian therapy, with a dose of long-acting synthetic glucocorticoids such as prednisolone taken at bedtime, i.v., and s.c. infusions of hydrocortisone to mimic the cortisol rhythm and more recently, the development of modified-release formulations of hydrocortisone.

Reverse circadian dosing is commonly used in adults with 21OHD-CAH. In two large cohort studies, >50% of patients were taking a glucocorticoid dose late at night (2, 3). Despite the different treatment regimens, optimal biochemical control, defined as 17OHP below three times the upper limit of normal (<36 nmol/L, <1200 ng/dL) was only achieved in <55% of patients. Reverse circadian dosing may improve control in patients, but it is evident from a pharmacokinetic study in patients with 21OHD-CAH that, despite taking prednisone and dexamethasone late at night, ACTH and 17OHP still show an exaggerated rise in the morning (38). There is also evidence that taking glucocorticoids later in the evening, when cortisol levels are normally low, may result in metabolically adverse consequences (39). Thus, reverse circadian dosing may improve control of androgens but has the downside of long-term excess exposure to glucocorticoids. Dexamethasone in particular has been associated with low bone mineral density and increased BMI (39) and prednisolone with increased mortality in replacement of patients with primary adrenal insufficiency compared to replacement with hydrocortisone (40).

The i.v. infusion of hydrocortisone via a pump can replicate the overnight rise in cortisol, and in two poorly controlled patients with 21OHD-CAH prevented the exaggerated rise in 17OHP within the first 24 h of pump therapy (41). Continuous s.c. hydrocortisone infusion by pump was trialled in a 6 month phase 2 study in eight poorly controlled patients with 21OHD-CAH (42). At study entry, all had elevated adrenal androgens and one or more comorbidities. The s.c. infusion approximated physiologic cortisol secretion and improved the control of 17OHP and A4 throughout the 24 h. This treatment was associated with clinical benefits including improved quality of life, restoration of menses in one of three amenorrhoeic women, and reduction of testicular adrenal rest tissue in one man. Five of the eight patients, however, had skin infections at the site of the infusion. Continuous s.c. infusion of hydrocortisone demonstrates that replicating the cortisol circadian rhythm of cortisol can improve the biochemical control of 21OHD-CAH and is associated with clinical benefit, but it is not a practical therapy for most patients.

Plenadren^®^ (Shire Services BVBA, Belgium) is a modified-release formulation of hydrocortisone licenced in Europe for the treatment of adrenal insufficiency. It is a dual-release tablet with an outer immediate-release hydrocortisone coating and an inner sustained-release hydrocortisone core. Taken once daily, it provides daytime replacement of hydrocortisone, but there is no overnight replacement of cortisol. Plenadren^®^ has been used in patients with 21OHD-CAH (43) but reported morning levels of 17OHP have been very elevated in most patients when taking Plenadren^®^, indicative of poor biochemical control (44). A clinical trial assessing its potential use in patients with 21OHD-CAH is ongoing (NCT03760835).

Efmody^®^ (development name Chronocort, Diurnal Europe B.V., The Netherlands) is a modified-release formulation of hydrocortisone licenced in Europe and Great Britain for the treatment of 21OHD-CAH in patients 12 years old and above. Chronocort is a multi-particulate formulation of hydrocortisone with a delayed-release coating that allows for delayed and sustained absorption. When taken at bedtime and on arising, Chronocort replicates the overnight diurnal rise in cortisol (45). In a phase 2 switch study, in 16 adult patients with 21OHD-CAH, Chronocort, at a lower dose than the standard treatment, improved control of 21OHD-CAH (46), and 94% of patients maintained good control (morning 17OHP <36 nmol/L, 1200 ng/dL) during Chronocort therapy.

**Figure 5:**
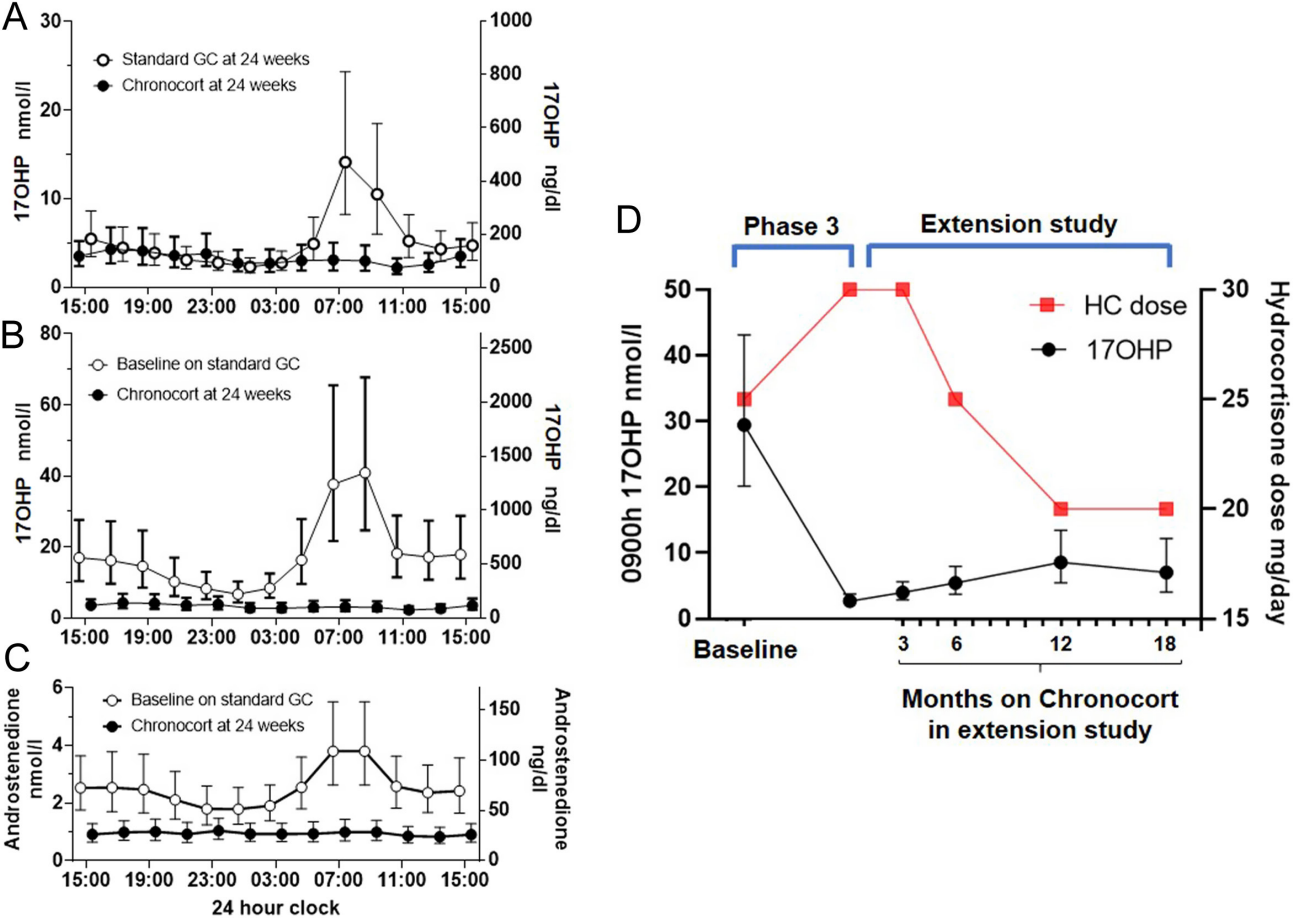
Chronocort phase 3 and extension study. (A) 24-h profile of 17OHP levels at 24 weeks comparing titrated standard treatment and Chronocort showing that Chronocort improves morning control of 17OHP. (B and C) 24-h profiles of 17OHP and A4 at baseline and after 24 weeks showing that Chronocort normalizes 17OHP levels and this is associated with low levels of A4. (D) 9:00 h 17OHP levels during Chronocort treatment from the phase 3 through the 18 months extension study compared to median hydrocortisone dose showing that during the extension study the hydrocortisone dose came down to a median dose of 20 mg and control of 17OHP was maintained (note right y axis represents current hydrocortisone dose range used in patients with CAH). Hormone levels are shown as geometric mean ± 95% CI (adapted from Merke et al. (47)).

In the phase 3 Chronocort study (47), 122 adult patients with 21OHD-CAH were randomized to continue either standard therapy or switch to Chronocort with a dose taken at bedtime and on arising. Standard therapy consisted of a variety of treatment regimens including hydrocortisone, prednisolone, and dexamethasone (both singly and in combination), and 84% of patients were taking standard glucocorticoids after 18:00 h in a reverse circadian fashion. Dose titrations were made for both treatment groups, using identical rules, by two independent physicians blinded to all data except the 24-h 17OHP and A4 profiles and an investigator-completed checklist screening for clinical signs and symptoms of possible glucocorticoid over- or under-treatment. Patients who enrolled in the phase 3 and the previous phase 2 studies were invited to enrol in an open-label extension study of Chronocort treatment (47). In the extension study, dose titration was performed by the local investigators as in a ‘real world’ experience. At the end of the phase 3 trial, patients who received Chronocort had superior hormonal control during the morning and early afternoon compared to those receiving standard therapy, and this advantage was sustained during 18 months follow-up (Fig. 4). The trial failed to meet its primary endpoint, because the difference between the two groups in the morning did not translate into a difference over 24 h at 6 months. The prespecified methods for data analysis obscured the impact of Chronocort in the morning and early afternoon (Fig. 5A). The raw data showed significant improvement of the clinically relevant endpoint of morning biochemical control, with reduced AUC and 17OHP amplitude in patients receiving Chronocort (Fig. 5A, B and  C). At baseline, in the phase 3 study, patients were taking a median hydrocortisone dose equivalent of 25 mg, and after 6 months, both groups had been titrated up to ~30 mg. In the extension study, where clinicians titrated the chronocort dose, the median daily dose fell from 30 to 20 mg over time (Fig. 5D). The improvement in biochemical control compared to standard treatment was maintained at 18 months, with 80% displaying good control (morning 17OHP < 36 nmol/L, <1200 ng/dL) for 17OHP and 96% for A4 (in the reference range) vs 52 and 45% at baseline, respectively. The improved disease control was observed despite a reduction in hydrocortisone dose by 33%, to doses typically used for adrenal replacement therapy (15–25 mg/day). Chronocort therapy was associated with patient-reported clinical benefit including menses restoration in eight patients (one on standard therapy), and three patient and four partner pregnancies (none on standard therapy); one of these male patients had a history of testicular adrenal rest tissue with documented sperm count improvement (<0.1 million/mL prior to Chronocort and 10.3 million/mL during Chronocort treatment). During the extension study, quality of life, which was good at baseline, was maintained, and no weight gain was observed. In the phase 3 study, no patients experienced adrenal crises in the Chronocort group compared with three in the standard group, and in the extension study, there were four patients with an adrenal crisis over 18 months. In the phase 3 study, no severe adverse event was considered related to the study intervention, and in the extension study, severe adverse events were reported for 14 participants one of which, hypokalaemia, was considered related to Chronocort.

The Chronocort development programme included the first randomized study of glucocorticoid therapy and afforded some key learning points. At entry into the phase 3 study, patients were relatively well controlled receiving standard treatment, but with intense monitoring, using 24-h hormone profiles and blinded titrators, control was improved with standard treatment that included long-acting glucocorticoids, with the majority of patients taking a bedtime dose of glucocorticoid. Nevertheless, the standard glucocorticoid treatment could not prevent the fluctuations and excessive rise of adrenal androgens seen in the morning, and the monitoring regime used in the study is not practical for routine clinical care. By delivering the pre-awakening rise in cortisol, Chronocort controlled the 17OHP into the optimal and, frequently, the reference range throughout the 24 h abolishing the fluctuations in 17OHP in most patients. At the end of the phase 3 study, the median daily dose of hydrocortisone equivalents was ~30 mg (15.8 and 17.0 mg/m^2^/day for Chronocort and standard group, respectively), similar doses to those reported in cohort studies (15–18 mg/m^2^/day) where biochemical control was worse than that in the Chronocort trial (2, 3, 48, 49). In the extension study, with local clinicians performing dose titrations, biochemical control was maintained at a reduced daily Chronocort dose of 20 mg consistent with that recommended for replacement in adrenal insufficiency; 15–25 mg daily (14). The maintenance of control despite dose reduction suggests that lower cortisol exposure is necessary when circadian replacement regimens are used chronically, perhaps related to a reduction in the size of the hyperplastic adrenals.

Another key learning point is that when 17OHP was well-controlled, A4 levels were low. This finding can be explained because not only is 17OHP an inefficient precursor of A4 but also the classic 17-hydroxypregnenolone-to-DHEA pathway is downregulated in 21OHD-CAH (38). This analysis is important, because, if the clinicians had depended on A4 to titrate patients, they would have reduced the dose of Chronocort below that required for adrenal replacement therapy. In the extension study, clinicians were able to titrate dosing using once or twice daily sampling during clinic times and independent of dosing, as 17OHP levels show little fluctuation while patients take Chronocort. Controlling the overnight rise in 17OHP with Chronocort also reduces the output of all adrenal androgen pathways including the 11-oxygenated androgen pathway and the alternative DHT pathway (50). The importance of controlling the fluctuations in 17OHP in the morning is shown by the patient-reported clinical benefit from Chronocort, with the restoration of menses and fertility in both men and women. In the extension study, 4 patients had an adrenal crisis with a frequency of 6.2 crises per 100 treatment years, similar to population estimates of 5–10 adrenal crises/100 patient-years (51, 52) and providing confidence that the safety profile of Chronocort does not differ from that of immediate-release hydrocortisone.

## Adrenal-targeted therapies

The defect in 21OHD-CAH is the deficiency of the adrenal enzyme, 21-hydroxylase, so it makes sense to target therapy at the adrenal steroidogenic pathway. Two drugs, abiraterone acetate (Janssen Research & Development, Raritan, NJ, USA) and Nevanimibe (Millendo Therapeutics, Ann Arbor, MI, USA), that block different levels of the adrenal steroidogenic pathway have been trialled in patients with 21OHD-CAH.

Abiraterone acetate is a prodrug, which is metabolized to abiraterone, a potent active site-directed inhibitor of CYP17A1 (17α-hydroxylase/17,20-lyase). Abiraterone acetate added to medical castration suppresses circulating testosterone and improves survival in castration-resistant prostate cancer (53). As androgen biosynthesis requires CYP17A1 activities, it is a rational approach to use Abiraterone acetate to control the androgen excess in 21OHD-CAH and obviate the need for supraphysiological glucocorticoids. In a phase 1, dose-escalation study, six adult females with 21OHD-CAH were administered Abiraterone acetate 100 or 250 mg every morning with 20 mg/day hydrocortisone for 6 days (54). With 100 mg/day Abiraterone, mean pre-dose A4 fell from 764 to 254 ng/dL (26.7–8.9 nmol/L). At 250 mg/day, mean A4 normalized in five participants (83%) and decreased from 664 to 126 ng/dL (23.2–4.4 nmol/L). Mean A4 declined further during day 6–66 and 38 ng/dL (2.3 and 1.3 nmol/L) at 100 and 250 mg/day, respectively. Hypertension, hypokalaemia, and peripheral oedema, secondary to 11-deoxycorticosterone accumulation are regularly seen in patients with prostate cancer treated with abiraterone but were not observed in patients with 21OHD-CAH because of the defective conversion of progesterone to 11-deoxycorticosterone. In addition to the potential benefit of lower glucocorticoid requirements, the reduction of androgen and oestrogen biosynthesis secondary to abiraterone could be beneficial in prepubertal children with 21OHD-CAH to normalize linear growth, a hypothesis currently being tested in clinical trial NCT02574910. Because of the effect on gonadal steroidogenesis and potential adverse fetal outcomes seen in preclinical studies, abiraterone is not suitable for chronic therapy in adult males or women of childbearing age.

Nevanimibe potently inhibits acyl-coenzyme A:cholesterol *O*-acyltransferase 1 (or sterol *O*-acyltransferase 1), the principal enzyme that catalyzes the esterification of free cholesterol to cholesteryl esters for storage in adrenal cortex cells. At lower concentrations, Nevanimibe reduces adrenal steroidogenesis across all three adrenocortical steroid pathways. In a phase 2 single-blind, dose titration study, *n*  = 10 adults with 21OHD-CAH poor disease control received the lowest dose of Nevanimibe (125 mg twice daily) for 2 weeks followed by a single-blind 2-week placebo washout (55). Nevanimibe was gradually titrated up (up to 1000 mg twice daily) if the primary outcome measure (17-OHP ≤ 2× ULN) was not met. Two subjects met the primary endpoint, and five others experienced 17-OHP decreases ranging from 27 to 72%. The most common side effects were gastrointestinal (30%), and one subject discontinued the study because of a severe adverse event (enteritis). Further development of Nevanimibe for 21OHD-CAH was halted after the interim review of a phase 2b study, which showed insufficient efficacy to continue the programme.

## Therapies targeting peripheral androgen action

Suboptimal treatment outcomes due to hyperandrogenism or glucocorticoid excess can potentially be addressed by giving physiological glucocorticoid replacement doses with antiandrogens. This strategy was tested in a 2-year trial of 28 children with 21OHD-CAH randomized to standard hydrocortisone therapy or to a combination of reduced hydrocortisone dose + flutamide (androgen receptor antagonist) + testolactone (aromatase inhibitor to prevent androgen-to-oestrogen conversion) (56). The combination therapy normalized growth and bone maturation, and the results of a long-term study testing the efficacy and safety of this treatment strategy are awaited (NCT00001521).

## Preclinical therapy development

Various other therapies are in preclinical development and include anti-ACTH monoclonal antibodies (57), ACTH receptor antagonists (58), gene-based therapy (59, 60, 61, 62), and cell-based therapy approaches (63). While most of these treatment strategies do not eliminate the need for glucocorticoid replacement, gene- and cell-based therapies have the anticipated hope that replacing the 21-hydroxylase enzyme itself will allow both replacement of cortisol and control of androgens (59). Adrenocortical-like, steroid-secreting cells have been generated from fibroblasts-, blood-, and urine-derived cells through cellular reprogramming and were viable when transplanted into the mouse adrenal gland or kidney capsule (63). Also, the impaired steroidogenesis of reprogrammed cells derived from patients with 21OHD-CAH was reversed through lentiviral delivery of the WT 21-hydroxylase-encoding gene (63). Gene-based therapy has also been tested and has shown promising results in temporarily reverting the 21OHD-CAH-like phenotype of 21-hydroxylase-deficient mice (59, 60, 61). Currently, there is a trial proposed for gene therapy to provide functional copies of the 21-hydroxylase-encoding gene using an adeno-associated virus (NCT04783181).

## Conclusions

The last 10 years have seen great advances in our understanding of the pathophysiology of 21OHD-CAH and with it a recognition that our current management is suboptimal. This impetus has led to a variety of different pharmacological approaches to improve the control of 21OHD-CAH. CRF1 receptor antagonists and adrenal enzyme blockers have shown they can reduce androgen biomarkers in patients with poorly controlled 21OHD-CAH, and further studies are planned to test whether they can improve health outcomes. Improved glucocorticoid delivery to provide accurate dosing in children and better replicate the circadian rhythm of cortisol either by infusion of hydrocortisone or treatment with Chronocort, a delayed-release formulation of hydrocortisone, can control androgen biomarkers using an adrenal replacement glucocorticoid dose, and patients report restoration of menses and pregnancies. The only treatments in development that have the potential to cure 21OHD-CAH and both control adrenal androgens and replace glucocorticoid treatment altogether are gene- and cell-based therapies.

## Declaration of interest

R J R is a Director of Diurnal Group Plc. R J A has received contracted research fees from Neurocrine Biosciences, Spruce Biosciences, and Millendo Therapeutics.

## Funding

This work did not receive any specific grant from any funding agency in the public, commercial, or not-for-profit sector.
